# Structural and optical properties of ITO/TiO_2_ anti-reflective films for solar cell applications

**DOI:** 10.1186/1556-276X-9-175

**Published:** 2014-04-11

**Authors:** Khuram Ali, Sohail A Khan, Mohd Zubir Mat Jafri

**Affiliations:** 1 Nano-Optoelectronics Research and Technology Laboratory, School of Physics, Universiti Sains Malaysia, Penang 11800, Malaysia

**Keywords:** ITO, TiO_2_, Sputtering, Antireflective films, Room temperature

## Abstract

Indium tin oxide (ITO) and titanium dioxide (TiO_2_) anti-reflective coatings (ARCs) were deposited on a (100) P-type monocrystalline Si substrate by a radio-frequency (RF) magnetron sputtering. Polycrystalline ITO and anatase TiO_2_ films were obtained at room temperature (RT). The thickness of ITO (60 to 64 nm) and TiO_2_ (55 to 60 nm) films was optimized, considering the optical response in the 400- to 1,000-nm wavelength range. The deposited films were characterized by X-ray diffraction (XRD), Raman spectroscopy, field emission scanning electron microscopy (FESEM), energy dispersive spectroscopy (EDS), and atomic force microscopy (AFM). The XRD analysis showed preferential orientation along (211) and (222) for ITO and (200) and (211) for TiO_2_ films. The XRD analysis showed that crystalline ITO/TiO_2_ films could be formed at RT. The crystallite strain measurements showed compressive strain for ITO and TiO_2_ films. The measured average optical reflectance was about 12% and 10% for the ITO and TiO_2_ ARCs, respectively.

## Background

To deposit titanium dioxide (TiO_2_) and indium tin oxide (ITO) films, several techniques have been used, including radio-frequency (RF) sputtering, chemical vapor deposition [1], sol–gel [2], spray deposition [3], and electron-beam evaporation [4]. Low-deposition temperatures are required because high temperatures can degrade a substrate material for solar cells and plastic devices [5]. RF sputtering is a sophisticated process with high deposition rate and good reproducibility [6]. Most of these techniques require a type of heat treatment (250°C to 650°C) for the substrates during or after the deposition [1,2,4], due to insufficient crystallization at RT. This phenomenon leads to poor optical and structural properties [7]. RT deposition is important for photovoltaic devices as the thermal treatments may change the intended compositional distribution and also introduce defects that act as recombination centers for charge carriers in the solar cell device. Many attempts have been made to deposit ITO and TiO_2_ thin films on silicon substrates by RF sputtering technique at RT [8,9]. The ITO film exhibits excellent conductivity and it can be used as an ohmic contact on a p-type c-Si. De Cesare, et al. achieved good electrical properties with ITO/c-Si contact at RT [10]. ITO has also become the attractive material for its anti-reflection (AR) properties and enhanced relative spectral response in the blue-visible region. Optical device performance depends greatly on the surface morphology and crystalline quality of the semiconductor layer [11].

Another material, TiO_2_, is well known in silicon processing technology and has wide applications in optics and optoelectronics [12,13]. TiO_2_ films can be distinguished into three major polymorphs: anatase, rutile, and brookite. Each phase exhibits a different crystal configuration with unique electrical, optical, and physical properties. Anatase is the most photoactive but thermally instable and it converts into rutile phase above 600°C [14,15]. In this paper, RF sputtering of ITO/TiO_2_ is used to eliminate the standard high-temperature deposition process required for the formation of AR films. This also guarantees that the critical surface layer of the monocrystalline Si is not damaged. Present work reports the crystal structure, optical reflectance, and microstructure of the ITO/TiO_2_ AR films, RF sputter deposited on monocrystalline Si p-type (100) at RT.

## Methods

ITO and TiO_2_ were deposited on a 0.01- to 1.5-Ω cm boron-doped monocrystalline Si wafer with one side polished. Silicon substrates were cleaned by a standard Radio Corporation of America method to remove surface contamination. After rinsing with deionized water (*ρ* > 18.2 MΩ cm) and N_2_ blowing, the ITO and TiO_2_ layers were deposited onto the front side of silicon wafers by RF sputtering using an Auto HHV500 sputtering unit. Table 1 shows the sputtering conditions for ITO and TiO_2_ films. The thickness of the single-layer ITO and TiO_2_ films was deduced from the following relation:

(1) $$d=\frac{\lambda_{o}}{4n}$$

where *λ*_o_ is the mid-range wavelength of 500 nm and *n* and *d* are the refractive index and film thickness, respectively. The morphology of the ITO and TiO_2_ films was characterized by atomic force microscope (AFM; Dimension Edge, Bruker, Santa Barbara, CA, USA). To determine the crystallite structure of films, X-ray diffraction (XRD) measurements were carried out using a high-resolution X-ray diffractometer (PANalytical X'pert PRO MRD PW3040, Almelo, The Netherlands) with CuKα radiation at 0.15406-nm wavelength. The surface reflectivity of films and reference p-type (100) were measured using a Filmetrics F20 optical reflectometer using white light within the frequency range of 3 × 10^14^ to 7.5 × 10^14^ Hz. The combined wavelengths ranged from 400 to 1,000 nm with different colors. Raman studies were carried out using a spectroscopy system (Jobin Yvon HR 800 UV, Edison, NJ, USA).

**Table 1:** **The growth parameters and results of the ITO and TiO**_**2 **_**film deposition on the Si substrate**

**Target**	**ITO 99.99%**	**TiO**_**2 **_**99.99%**
Target diameter	7.6 cm	7.6 cm
Distance from substrate	10 cm	10 cm
Substrate	Si	Si
Substrate temperature	30°C	35°C
Ultimate pressure	2.68 × 10^-5^ mbar	2.97 × 10^-5^ mbar
Vacuum (plasma) pressure	7.41 × 10^-3^ mbar	6.75 × 10^-3^ mbar
Gas	Ar 99.99%	Ar 99.99%
RF sputtering power	200 W	200 W
Deposition rate	2.1 Å · s^-1^	0.5 Å · s^-1^
Deposition time	5 min	19 min
Required thickness	60 to 64 nm	55 to 60 nm
Crystalline size	0.229 nm	0.223 nm
*n* (*λ* = 500 nm)	1.97	2.2

## Results and discussion

**Figure 1:**
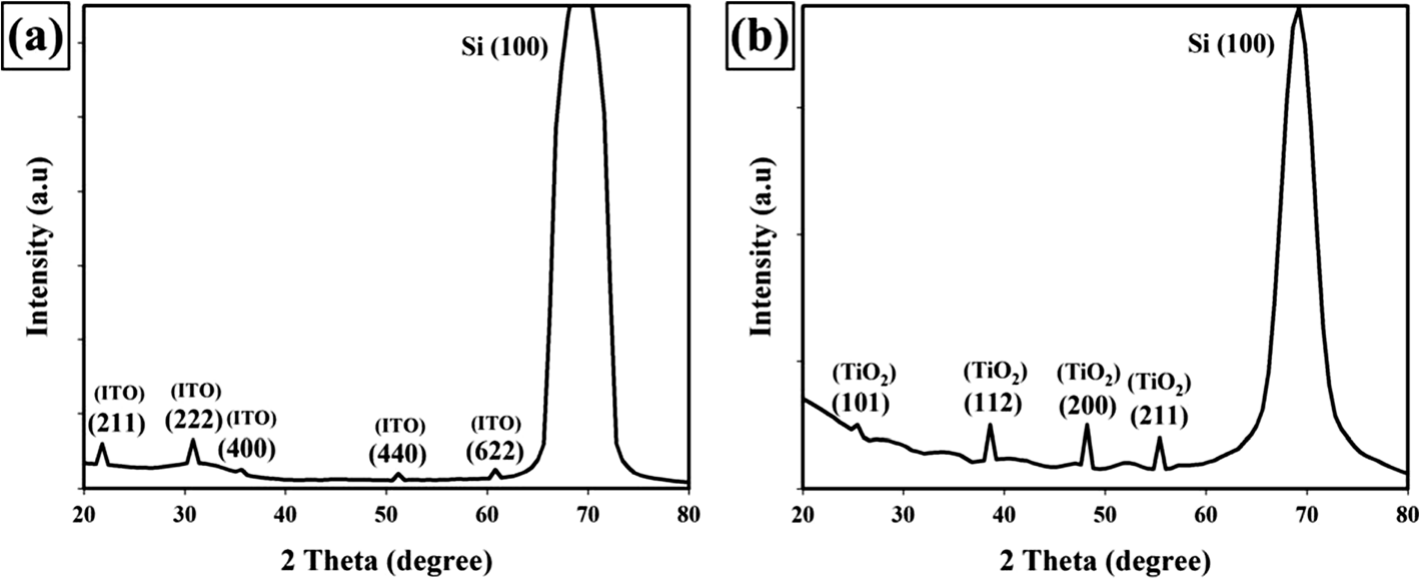
**XRD spectrum of (a) ITO and (b) TiO**_**2 **_**films.**

Typical XRD measurements of ITO films deposited by RF magnetron sputtering at RT are represented in Figure 1a. The low-intensity diffraction peak analogous to an incipient crystallization of the ITO in the (222)-oriented body-centered cubic (bcc) structure has been identified. While other diffraction peaks such as (400), (440), (611), and (622) showing crystallites with other orientation. The reflection from the (2 2 2) crystalline plane resulted in a characteristic peak at 2*θ* = 30.81°, which was close to the peak (2*θ* = 30.581°) of the reference ITO [11,16,17]. The structural and morphological characteristics of the ITO film showed polycrystalline ITO growth on Si p-type (100) at RT [18].

Figure 1b shows the XRD patterns of the TiO_2_ film grown on Si (100) substrates at RT. All diffraction peaks at 25.42°, 38.60°, 48.12°, and 55.39° corresponded to anatase (1 0 1), (1 1 2), (2 0 0), and (2 1 1) crystal planes, respectively [14,15]. The result of the XRD patterns also showed that the anatase (2 0 0) is the preferential growth orientation while no rutile phases were observed. Anatase phase of TiO_2_ film grown on Si p-type (100) at RT is highly photoactive and have better AR properties as compared to other TiO_2_ polymorphs: rutile and brookite [19]. XRD measurements affirm that nanocrystalline TiO_2_ film with the anatase phase could be grown at RT without any apparent contamination. Table 1 lists the average crystallite size calculated using the Scherrer formula in Equation 2 [20].

(2) $$D=\frac{0.9\lambda }{\beta \text{cos}\theta }$$

where *D* is the average crystallite size, *λ* is the X-ray radiation wavelength (0.15406 nm), *β* is the full width at half maximum (FWHM) value, and *θ* is the diffraction Bragg angle.

**Figure 2:**
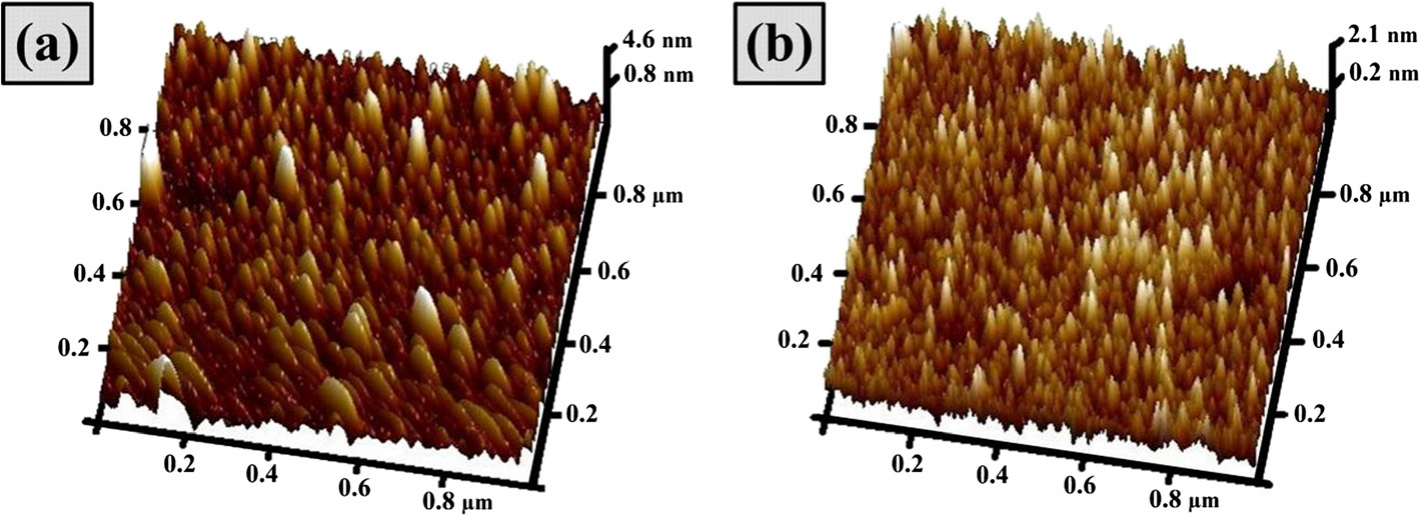
**AFM images of (a) ITO and (b) TiO**_**2 **_**films.**

The film microstructure of ITO and TiO_2_ films was also investigated by AFM, and the results are shown in Figure 2. Typical morphological features can be perceived readily by visual inspection of Figure 2a,b. As can be seen, the granules of different scales exist in both the films and are scattered evenly in some ranges. In quantitative analyses on AFM images, surface morphology can be described by using the height roughness (Ra) and root mean square roughness (rms). Ra is described as the mean value of the surface height analogous to the center plane while rms is the standard deviation of the surface height within the given area [11]. From Figure 2a, height roughness (Ra) and root mean square roughness (rms) values of 0.75 and 9.4 nm, respectively, were determined for the surface roughness of ITO film deposited at RT. While from Figure 2b, Ra and rms values of 0.39 and 6.9 nm, respectively, were determined for the surface roughness of TiO_2_ film deposited at RT. The above analysis indicates that Ra and rms are strongly affected by the degree of accumulation and cluster size of the films.

**Figure 3:**
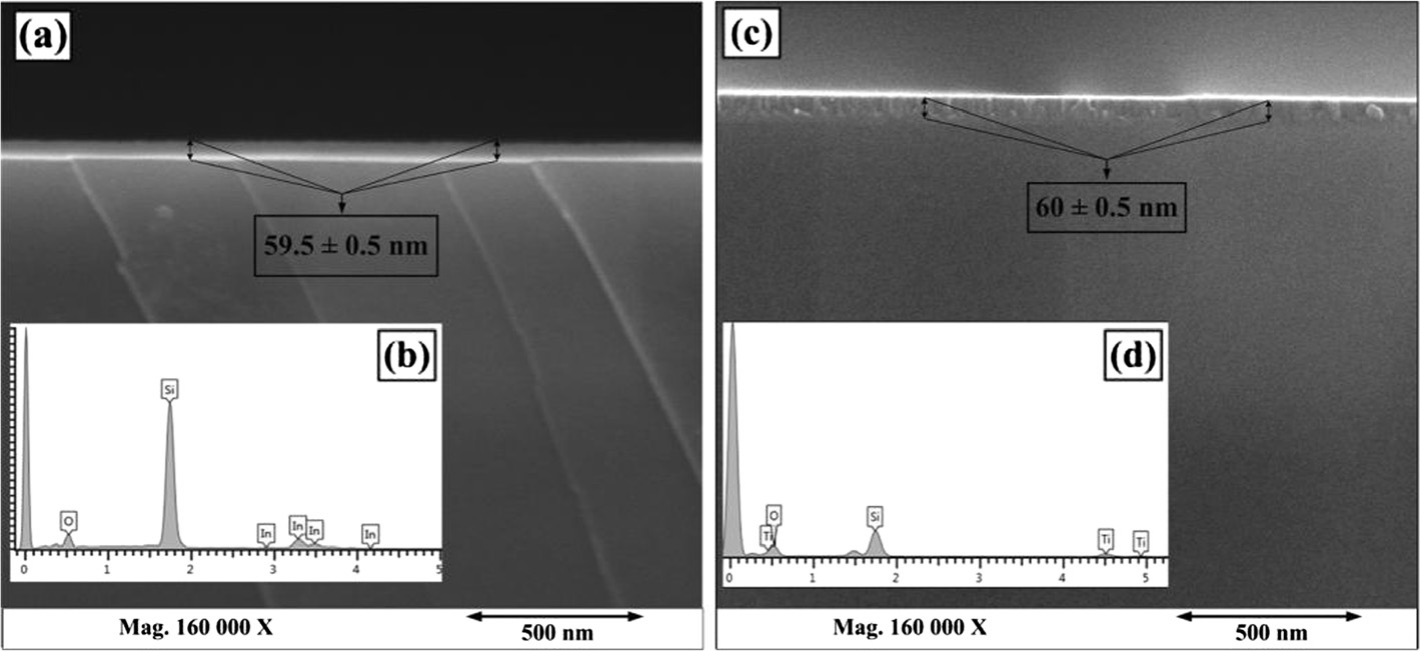
**FESEM cross-sectional view and EDX spectra of (a,b) ITO and (c,d) TiO**_**2 **_**films.**

**Figure 4:**
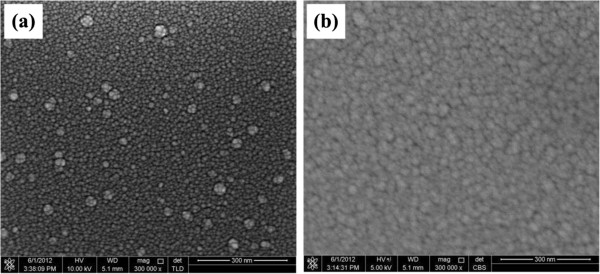
**FESEM images of front views of (a) ITO and (b) TiO**_**2 **_**films.**

Cross-sectional view of ITO and TiO_2_ films and respective energy dispersive X-ray (EDX) spectroscopy spectra are shown in Figure 3. FESEM cross-sectional view shows that the thickness of ITO and TiO_2_ films was 59.5 and 60 nm, respectively, with an average ±0.5 nm uncertainty in thickness. FESEM front view of ITO and TiO_2_ films is shown in Figure 4. Visual inspection of front view represents that the granules of various scales were uniformly distributed in both ITO and TiO_2_ films. These different scale granules influence the surface morphology of the films.

**Figure 5:**
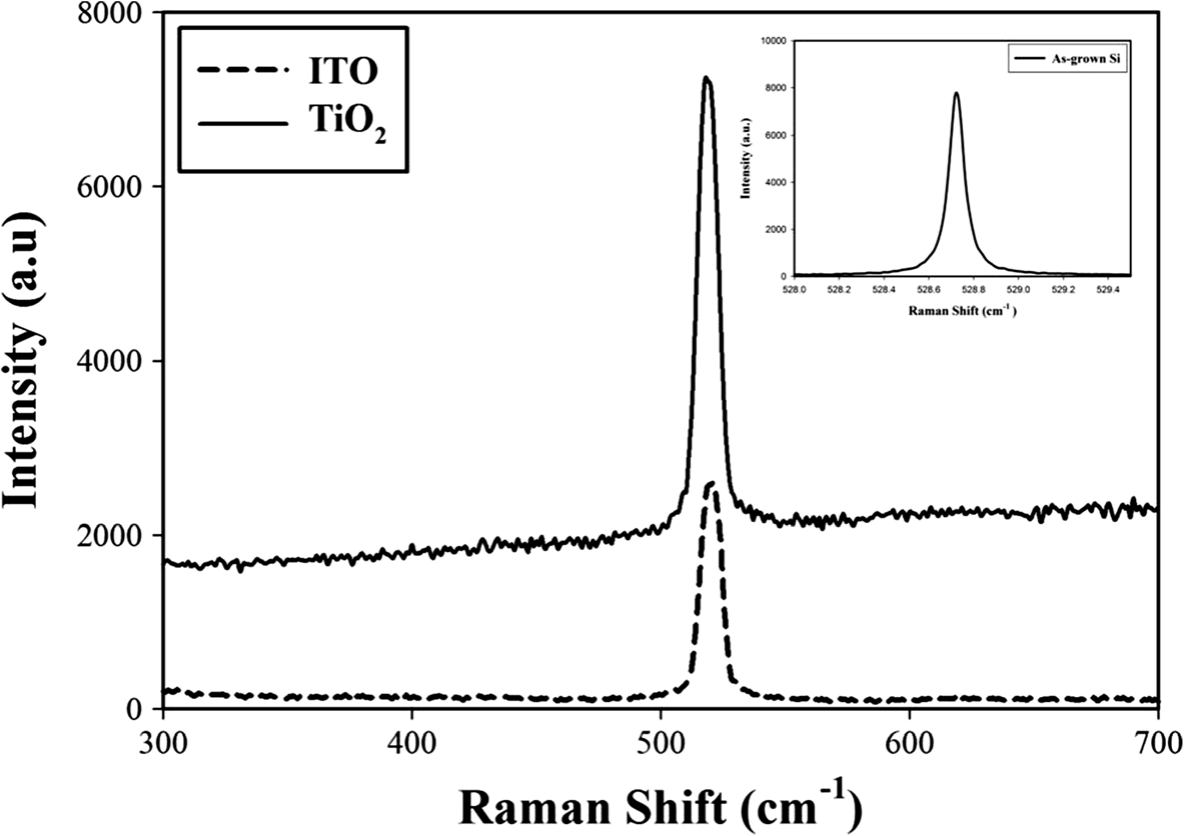
**Raman spectra of ITO and TiO**_**2 **_**films with the as-grown Si sample.**

Figure 5 shows the Raman spectra of the ITO films, TiO_2_ films, and as-grown Si sample based on the crystalline silicon p-type (100) at RT. Raman spectroscopy explains the structural changes pertinent to the strain within the films. The Raman spectra of the as-grown Si sample showed a sharp solid line with an FWHM of only 0.08 cm^-1^ located at 528.72 cm^-1^ because of the scattering of first-order phonons. The formation of the TiO_2_ layer led to a peak shift at 519.52 cm^-1^ with an FWHM of 10.24 cm^-1^, and to increased peak intensity compared with that of the ITO film and as-grown Si sample. The Raman spectra of the ITO layer shifted and sharpened at 518.81 cm^-1^ with an FWHM of 9.76 cm^-1^, and led to an increased peak intensity compared with that of the as-grown Si sample. The preferential growth on Si was characterized by considerable shifting in the peak position. These UV peaks were due to the near band edge emission and heterogeneous properties of both the films. The Raman spectra revealed blue shifts in both film peaks. It is known that the blue shift of the peak attributed to the residual compressive strain [21,22]. This result can be attributed to the quantum confinement of optical phonons in the electronic wave function of the Si nanocrystals.

**Figure 6:**
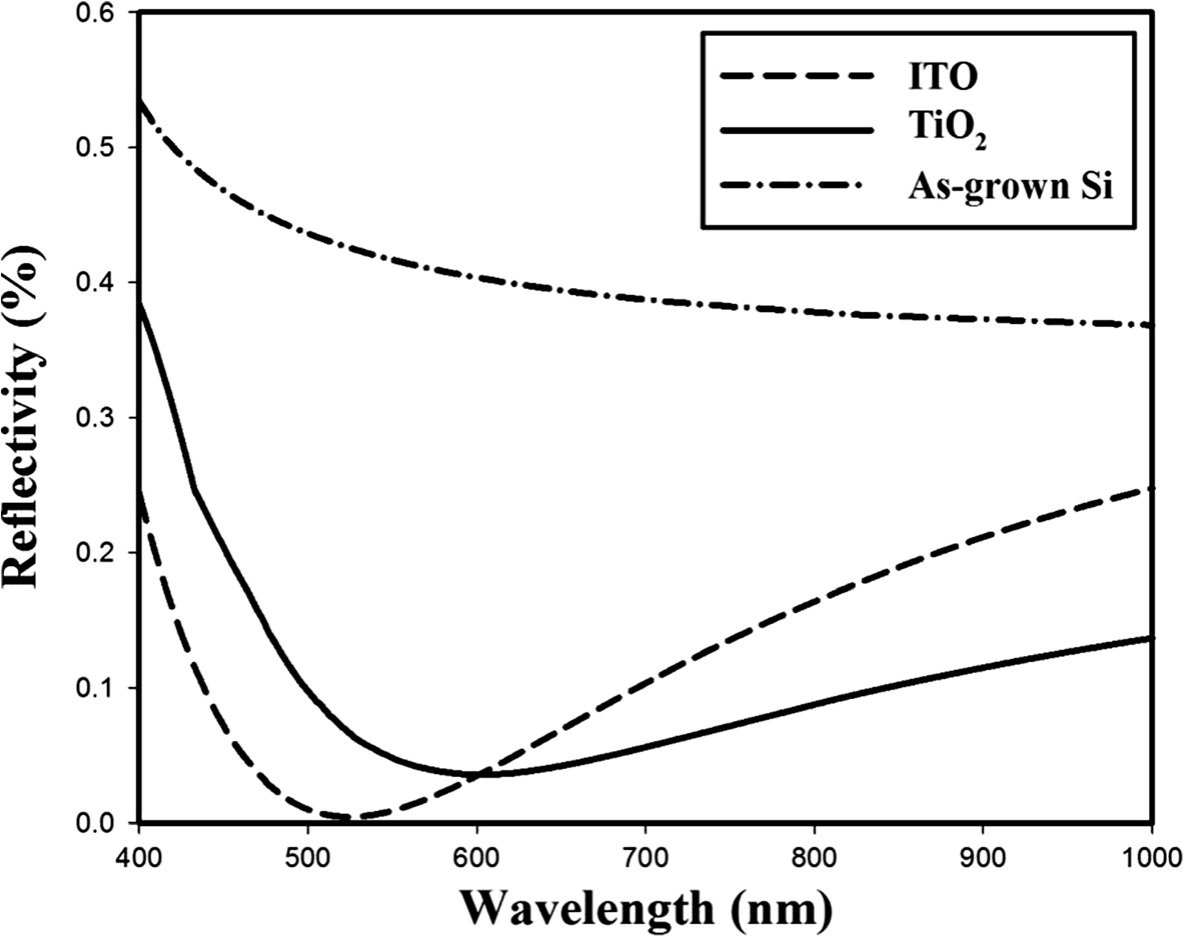
**Reflectance spectra for ITO and TiO**_**2 **_**layers with the as-grown Si sample.**

Figure 6 shows the measured reflectance spectra of ITO and TiO_2_ layers with the as-grown Si sample on non-textured Si substrates. The average solar reflectance of bare silicon was about 35%, whereas 10% and 12% were obtained by the deposition of TiO_2_ and ITO films, respectively, on the non-textured Si substrates. Reflection spectrum of ITO shows the minimum reflection of 0.4% at 523 nm while reflection spectrum of TiO_2_ shows the minimum reflection of 3.5% at 601 nm within the 400- to 1,000-nm range. It means the Si absorbance increased by approximately 25% and 23% for ITO and TiO_2_ films, respectively. The low reflectance enhances the absorption of the incident photons and hence increases the photo-generated current in Si solar cells. It reveals that the RT RF sputtering deposition of ITO and TiO_2_ films can be used as anti-reflective coatings (ARCs) for Si solar cells.

## Conclusions

The work presents the structural and optical characteristics of ITO and TiO_2_ ARCs deposited on a (100) P-type monocrystalline Si substrate by a RF magnetron sputtering at RT. X-ray diffraction proved the anatase TiO_2_ and polycrystalline ITO films structure. Residual compressive strain was confirmed from the Raman analysis of the ITO and TiO_2_ films which exhibited blue shifts in peaks at 518.81 and 519.52 cm^-1^ excitation wavelengths, respectively.

FESEM micrographs showed that the granules of various scales are uniformly distributed in both ITO and TiO_2_ films. Reflectance measurements of ITO and TiO_2_ films showed 25% and 23% improvement in the absorbance of incident light as compared to the as-grown Si. Low reflectivity value of 10% in the ITO film as compared to 12% of the TiO_2_ film is attributed to the high rms value. Our results reveal that the highly absorbent polycrystalline ITO and photoactive anatase TiO_2_ can be obtained by RF magnetron sputtering at room temperature. Both ITO and TiO_2_ films can be used as ARCs in the fabrication of silicon solar cells.

## Competing interests

The authors declare that they have no competing interests.

## Authors’ contributions

KA carried out the fabrication and characterization of the study and drafted the manuscript. SAK participated in its design and coordination and helped to draft the manuscript. MZMJ participated in the design and coordination of the study. All authors read and approved the final manuscript.
